# Protein Acetylation Increased Risk of Fibrosis-Related Liver Cancer

**DOI:** 10.1155/2023/3624635

**Published:** 2023-01-23

**Authors:** Yuan Li, Yanyan Wang, Zhaopu Song, Kai Lu, Wenwen Chen, Yuanyuan Ma, Hui Ding, Xiaofang Li, Xiuling Li, Suofeng Sun

**Affiliations:** ^1^Department of Traditional Chinese Medicine, The Third Affiliated Hospital Affiliated of Henan University of Traditional Chinese Medicine, Zhengzhou, Henan 450003, China; ^2^Department of Gastroenterology, Zhengzhou University People's Hospital, Henan Provincial People's Hospital, Zhengzhou, Henan 450003, China; ^3^Ruzhou Jingeng Rehabilitation Hospital, Ruzhou 467500, Henan, China; ^4^Xinxiang Medical University, Xinxiang 453000, Henan, China

## Abstract

**Objective:**

The occurrence of liver fibrosis and fibrosis-related liver cancer is the reason for the increase in morbidity and mortality worldwide. Transforming growth factor-*β*2 (TGF-*β*2) is an important mediator of chronic liver fibrosis. This study aims to find the molecular mechanism that mediates HBV infection and induces TGF-*β*2 and verifies that CREB binding protein acetylation mediates HBV infection and induces TGF-*β*2 expression.

**Methods:**

The acetylated proteins were extracted from HepG2-NTCP cells and HBV-infectedHepG2-NTCP cells. The acetylated proteins were screened by modification enrichment technology and database search. Protein annotation, motif analysis of modification sites, and protein function enrichment analysis of these proteins were performed to roughly clarify the location and function of these acetylated modification proteins in cells. Acylated proteins enriched in the TGF-*β* pathway were obtained by KEGG pathway enrichment analysis. The effect of the selected acetylated modification protein on the TGF-*β* pathway was verified by experiments, that is, the target protein gene was knocked out by siRNA, and the expression level of the TGF-*β*2 was detected by qRT-PCR.

**Results:**

Proteins were extracted from HepG2-NTCP cells and HepG2-NTCP cells infected with HBV, and differential acetylation modification proteins were screened. The target protein CREB binding protein was screened by modification enrichment technology and database search. The aggregation analysis of TGF-*β* pathway showed that CREB binding protein was acetylated at amino acid positions 434 and 439, and enriched in the TGF-*β* signaling pathway. siRNA targeting CREB binding protein was transfected, and the expression of TGF-*β*2 in cells was detected by qRT-PCR and western blot, respectively. It was verified that HBV infection-inducedCREB-binding protein acetylation regulated the high expression of TGF-*β*2.

**Conclusion:**

After HBV infection, CREBBP acetylation was up-regulated, which promoted the high expression of TGF-*β*2.

## 1. Introduction

Liver fibrosis can develop into chronic hepatitis and cancer of liver and was the main incentive of various liver diseases [1]. Progressive liver fibrosis and tumors have many causes, including non-alcoholic fatty liver disease, viral hepatitis, alcoholism, autoimmune hepatitis, nonalcoholic steatohepatitis (NASH), and biliary tract diseases [2]. Liver fibrosis was closely related to cancer. The incidence of hepatocellular carcinoma caused by liver cirrhosis is as high as 90%, which makes cirrhosis a major risk factor for liver cancer. The only effective treatment of liver fibrosis was eliminating irritation or liver transplantation. It was necessary to become potential antiliver fibrosis treatment methods to reduce fibrosis and the risk of liver cancer [3].

TGF-*β*2 always exists in all stages of the hepatic lesion [4]. The initial stage of the lesion induces hepatocyte apoptosis and HSCs transdifferentiation into myofibroblasts after chronic injury. TGF-*β*2 also promotes HSC proliferation and maintains myofibroblast phenotype, which is the key to the formation of liver cirrhosis. TGF-*β* also plays an important role in hepatocellular carcinoma; it acts as a tumor suppressor at the early stage, but once tumor cells gain the ability to overcome their cytostatic response, it activates key tumor-promoting factors. The role of cytokines is conducive to malignant progression [5].

Various causes of liver disease lead to liver fibrosis through a comprehensive signal network that regulates the deposition of extracellular matrix. The hepatitis B virus (HBV) is still the leading cause of liver fibrosis in China [6]. More and more evidence indicates that HBV infection may promote the production of transforming growth factor-*β* in liver cells, which in turn activates hepatic stellate cells and accelerates liver fibrosis [7, 8]. TGF-*β* is mainly produced by activated macrophages in the liver, which stimulates the activation of hepatic stellate cells (HSCs) into a myofibroblast-like phenotype [9, 10], promotes the differentiation of myofibroblasts, and stimulates the synthesis of extracellular matrix and down-regulating the degradation of extracellular matrix [11]. It is reported that TGF-*β*2 binds to the type II receptor on the cell surface, then recruits the type I receptor. TGF-*β*I receptor activates Smad2 and Smad3 proteins, and the activated Smad2 and Smad3 proteins specifically bind to each other. The binding protein complexes are phosphorylated and then bound to Smad4 proteins, and then transported to the nucleus to the binding to DNA, and regulate the transcription of extracellular matrix genes in the nucleus. In addition, studies have shown that Smad2 and Smad3 may also participate in the general transcription mechanism through direct interaction with p300 and CREBBP, and may participate in the development of liver fibrosis through the transcription of extracellular matrix [12].

**Figure 1:**
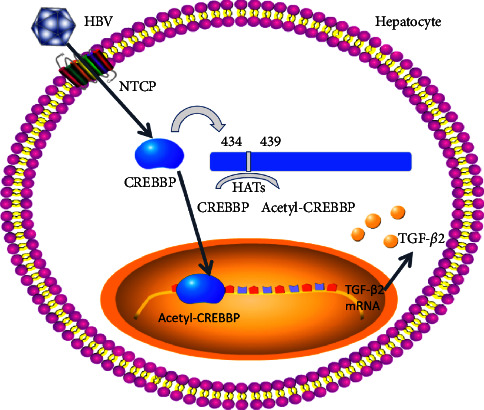
Schematic diagram of the signal transduction pathway. HBV infection up-regulates the acetylation level of CREB binding protein and induces high expression of TGF-*β*2.

The results have shown that CREB binding protein is involved in many cellular processes and functions. CREB binding protein interacts with a variety of transcription factors, including CREB binding protein and a variety of nuclear hormone receptors, to play the role of transcription co-activator and histone acetyltransferase [13, 14]. Studies have found that CREB binding protein is related to fibrosis, inhibiting Wnt/*β*-catenin/CREB binding protein signal transduction and reversing pulmonary fibrosis [15], Grap2 cyclin D interaction protein negatively regulates CREB binding protein and inhibits fibroblast-like synovial cell proliferation [13]. Currently, it was limited research on the role of CREB-binding protein in liver fibrosis. Inhibition of CREB binding protein/*β*-catenin can inhibit the formation of liver fibrosis and promote the regression of liver fibrosis [16]. In addition, CREB binding protein is involved in the signal pathway of hypoxia-induciblefactor-1*α* (HIF-1*α*), erythropoietin. These signaling pathways are activated during cerebral ischemia and exert neuroprotection [17]. Everyone has gradually realized the important relationship between TGF-*β* and fibrosis. Here, we have also proved that HBV infection up-regulates the acetylation level of CREB binding protein and induces high expression of TGF-*β*2 (Figure 1), which provides a new treatment for liver fibrosis.

Here, we report that the acetylation level of CREB binding protein is significantly up-regulated in HBV-infected hepatocytes and CREB binding protein mediates liver fibrosis through the TGF-*β* pathway.

## 2. Materials and Methods

### 2.1. Protein Preparation

The sample was sonicated three times on ice using a high-intensity ultrasonic processor (Scientz) in lysis buffer (8 M urea, 1% Protease Inhibitor Cocktail) (Note: For PTM experiments, inhibitors were also added to the lysis buffer, e.g., 3 *μ*M TSA and 50 mM NAM for acetylation). The remaining debris was removed by centrifugation at 12,000 g at 4°C for 10 min. Finally, the supernatant was collected and the protein concentration was determined with a BCA kit according to the manufacturer's instructions.

### 2.2. Protein Differential Modification Analysis

The dithiothreitol was added to the protein solution to a final concentration of 5 mM and reduced at 56°C for 30 min. Then add iodoacetamide to make the final concentration 11 mM, and incubate for 15 min at room temperature in the dark. Finally, the urea concentration of the sample is diluted to less than 2 M. Add pancreatin at a mass ratio of 1 : 50 (pancreatin: protein) and digest overnight at 37°C. Then add pancreatin at a mass ratio of 1 : 100 (pancreatin: protein) and continue enzymatic hydrolysis for 4 h. The signal abundance corresponding to the modified site in each sample was detected by mass spectrometry [18]. According to the strength of the modified peptide, strength of the modified peptide in each sample was obtained by nonstandard quantitative calculation.

### 2.3. Modification and Enrichment

Dissolve the peptide in IP buffer solution (100 mM NaCl, 1 mM EDTA, 50 mM Tris-HCl, 0.5% NP-40, pH 8.0), transfer the supernatant to the acetylated resin that has been washed in advance medium (antibody resin item number PTM104, from Hangzhou Jingjie Biotechnology Co., Ltd., PTM Bio), placed on a 4°C rotating shaker, gently shake and incubate overnight. After the incubation, the resin was washed 4 times with IP buffer solution and twice with deionized water. Finally, use 0.1% trifluoroacetic acid eluent to elute the resin-bound peptides, eluting three times, collecting the eluent, and vacuum freeze and drain. After draining, follow the instructions of C18 ZipTips for desalination, vacuum freeze draining, and then use for LC/MS analysis.

### 2.4. Motif Analysis

Soft MoMo (motif-x algorithm) was used to analyze the model of sequences constituted with amino acids in specific positions of modify-21-mers (10 amino acids upstream and downstream of the site, but phosphorylation with modify-13-mers that 6 amino acids upstream and downstream of the site) in all protein sequences. And all the database protein sequences were used as background database parameters. The minimum number of occurrences was set to 20. Emulate original motif-x was ticked, and other parameters with default.

### 2.5. Histological Analysis and Immunohistochemistry

Paraffin sections of human liver tissues were prepared by hematoxylin-eosin (H & E) staining. The paraffin sections were subjected to immunohistochemical detection of TGF-*β*2 and observed under a microscope (Olympus BX51, Japan). Image J software was used to quantitatively analyze TGF-*β*2.

## 3. Results

### 3.1. Sample Repeatability Test

**Figure 2:**
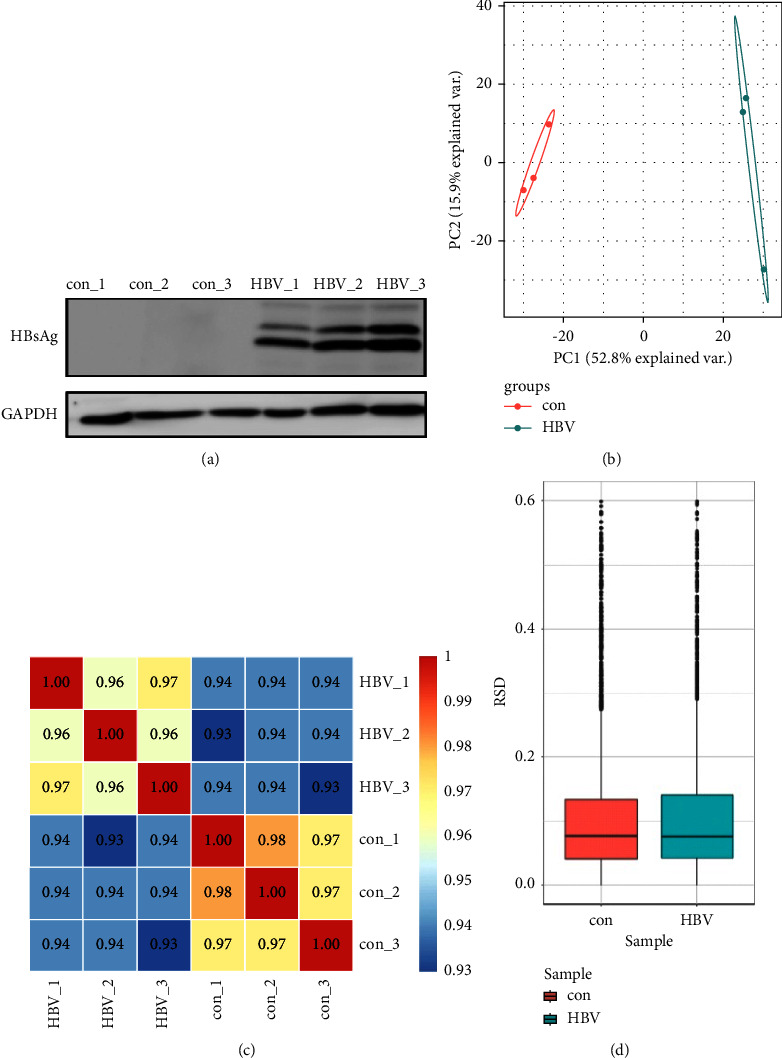
Using immunofluorescence and sample repeatability experiments to verify the validity of the experiment. (a) Con. 1–3 are the untransfected HBV group and HBV.1-3 are the transfected HBV group. (b) Principal component analysis (PCA). (c) A heat map drawn by calculating Pearson's correlation coefficient between all samples. (d) RSD of the modified quantitative value among replicate samples.

Repeatability experiments were used to verify the validity of the experiment. Compared with the control group, the experimental group was transfected with HBV-expressed HBsAg (Figure 2(a)). The effectiveness of this experiment is proved by using three statistical analysis methods: principal component analysis(Figure 2(b)), Pearson correlation (Figure 2(c)), and relative standard deviation (Figure 2(d)).

### 3.2. Analysis of Acetylated Modified Proteins

**Figure 3:**
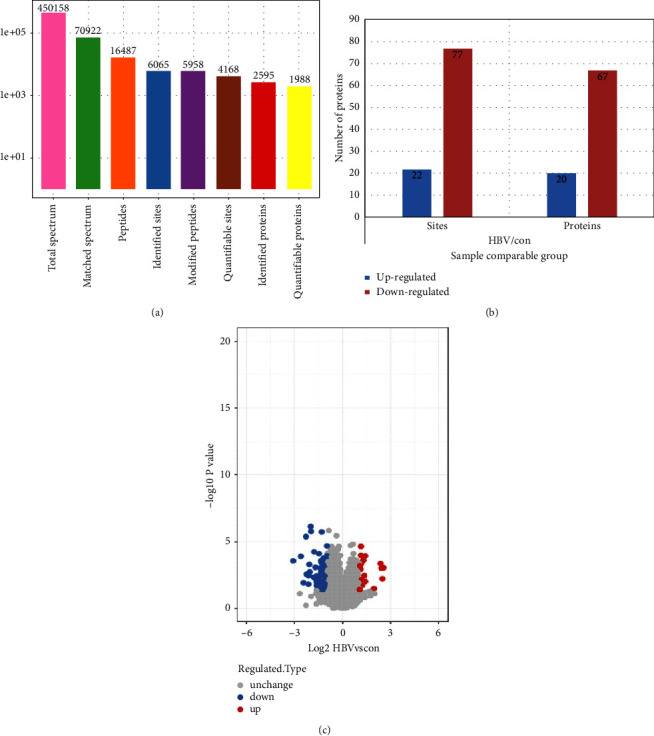
Screening of acetylated modified proteins through protein motif analysis of the proteins expressed by hepatocytes after HBV infection. (a) Statistical graph of the results of mass spectrometry data. (b) Columnar distribution of the number of modified proteins and modified sites. (c) Volcano plot of differentially modified site.

Through protein motif analysis of the proteins expressed by hepatocytes after HBV infection, the acetylated modified proteins were screened. Totally, 450158 secondary spectra were got by mass spectrometry. The secondary spectra of MS were retrieved from the protein theory database. The number of available effective spectra was 70922, and the utilization rate of spectra was 15.8%. A total of 16487 peptides and 5958 acetylated peptides were identified by spectral analysis. We have identified 6065.0 acetylation modification sites on 2595.0 proteins, of which 4168.0 sites on 1988.0 proteins have quantitative information (Figure 3(a)). Through protein differential modification analysis, 22 acetylation sites were up-regulated in HBV-transfected liver cancer cells, 77 acetylation sites were down-regulated, and 20 acetylation-modified proteins were up-regulated. The levels of two acetylated modified proteins were down-regulated (Figure 3(b)). Figure 2(c) shows the distribution of these differential proteins in the volcano map of differential modification sites (Figure 3(c)).

**Figure 4:**
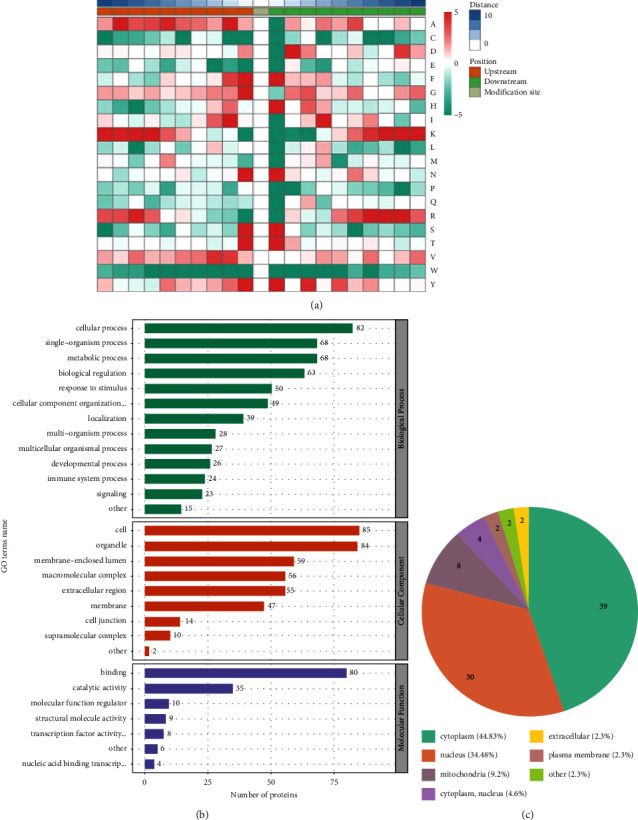
Analysis of acetylated modified proteins. (a) Heat maps of all upstream and downstream amino acids of the identified acetylation modification sites. (b) The statistical distribution map of the protein corresponding to the differential acetylation modification site in the GO secondary classification. (c) The subcellular structure location distribution map of the protein corresponding to the differential acetylation modification site.

Protein motif analysis calculates the regular trend of the amino acid sequence in the region where the acetylation modification site occurs by counting the rules of the amino acid sequence before and after all acetylation modification sites in the sample. This analysis can find the sequence characteristics of modification sites, and thus speculate or determine the enzymes related to modification (Figure 4(a)). In addition, gene ontology also explains the biological effects of proteins from three perspectives: biological process, molecular function, and cell composition. Most of the proteins corresponding to acetylation modification sites are distributed in cells and participate in cellular processes. Exerting molecular functions such as binding, structural molecular activity, transcription factor activity, catalytic activity, and molecular function modifier (Figure 4(b)). Among them, 39 proteins were distributed in the cytoplasm, accounting for 44.83%, 34.48% in the nucleus, and the rest in mitochondria, plasma membrane, extracellular, and so on (Figure 4(c)).

### 3.3. Functional Enrichment Analysis of Proteins Corresponding to Different Acetylation Modification Sites

**Figure 5:**
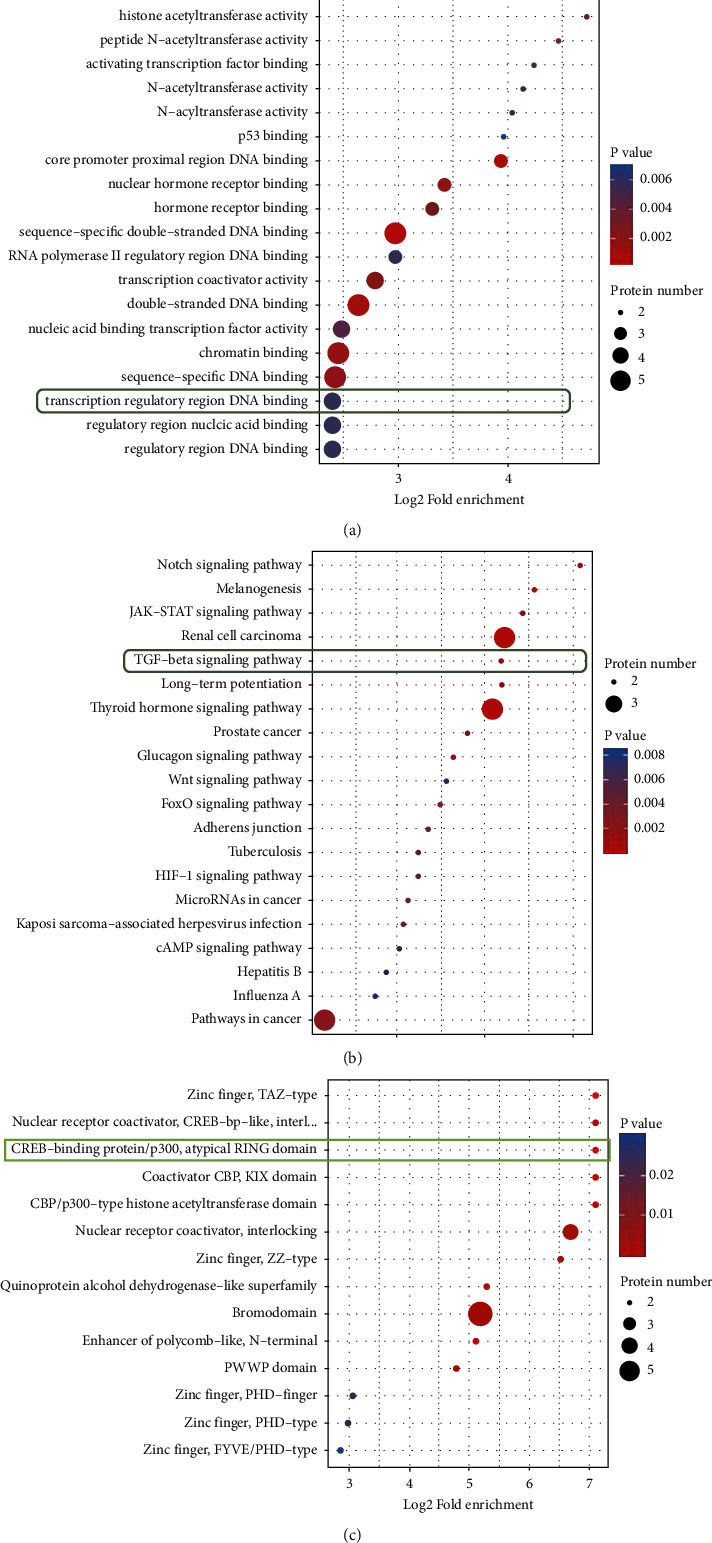
Functional enrichment analysis of proteins corresponding to different acetylation modification sites. (a) Differential acetylation sites corresponding protein GO enrichment distribution bubble diagram. (b) Bubble pattern of enrichment and distribution of proteins corresponding to differential acetylation modification sites in the KEGG pathway. (c) Enrichment and distribution of bubbles in protein domain classification corresponding to differential acetylation modification sites.

The bubble chart of the enrichment distribution in the GO classification shows that the differential protein has acetyltransferase activity, hormone receptor activity, and DNA binding function in the transcription regulatory region (Figure 5(a)) (protein function: peptide-lysine-N-acetyltransferase activity, histone acetyltransferase activity, peptide-N-acetyltransferase activity, activating transcription factor activity, N-acetyltransferase activity, N-acyltransferase activity, p53 binding, core promoter proximal DNA binding, nuclear hormone receptor binding, hormone receptor binding, sequence-specificdouble-stranded DNA binding, RNA polymerase II regulatory region DNA binding, transcription coactivator activity, double-stranded DNA binding, nucleic acid binding transcription factor activity, chromatin binding, sequence specific DNA binding, transcription regulatory region DNA binding, nucleic acid binding regulatory region, regulatory region DNA binding). The enrichment distribution of the proteins corresponding to the differential acetylation modification sites in the KEGG pathway shows that two interesting proteins are enriched in the TGF-bate signaling pathway (*P* < 0.002) (Figure 5(b)). Differential acetylation modification sites correspond to protein enrichment distribution in protein domain classification. The bubble chart shows that differential proteins are enriched in CBP/p300 atypical loop domains and CBP/p300 histone acetyltransferase domains (Figure 5(c)).

### 3.4. Cluster Analysis of Proteins Corresponding to Differential Acetylation Modification Sites

**Figure 6:**
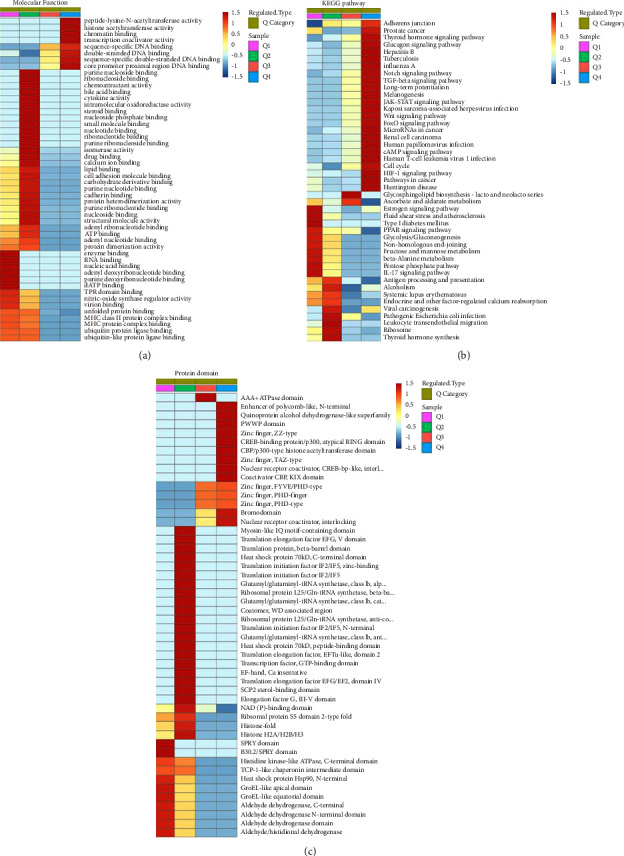
Cluster analysis of proteins corresponding to differential acetylation modification sites. (a)–(c) Clustering analysis heat map based on GO classification, KEGG pathway, and protein domain enrichment.

According to its differential modification multiples, it is divided into 4 parts, called Q1 to Q4. Q1 (<0.500), Q2 (0.500∼0.677), Q3 (1.5∼2.0), and Q4 (>2.0). Differential proteins are strongly enriched in the “transcriptional coactivator activity” molecular functions in the cluster analysis heat map of GO classification (Figure 6(a)) and in the TGF-*β* signaling pathway of the KEGG pathway (Figure 6(b)). The differentially modified proteins are enriched in the CBP/p300 atypical loop domain and CBP/p300 histone acetyltransferase domain (Figure 6(c)), and the differential modification multiples are all greater than 2.0.

In conclusion, acetylated proteins were differentially expressed in HBV-infected HCC cells, and the corresponding proteins at different modification sites were highly enriched in the TGF-*β* signaling pathway. In the domain classification of differential modification proteins, the strong aggregation was concentrated in the CBP/p300 atypical cyclic domain; differentially modified proteins have transcriptional regulatory functions.

### 3.5. Enrichment of Differentially Acetylated Protein CREBBP in TGF-*β* Pathway

CREB binding protein is enriched in the TGF-*β* signaling pathway. Further enrichment analysis of the TGF-*β* signal transduction pathway, as shown in Table 1, the CREB binding protein was acetylated at amino acid positions 434 and 439 and was enriched in the TGF-*β* signal transduction pathway.

### 3.6. HBV Infection Up-Regulates CREBBP Expression and Induces High Expression of TGF-*β*2

**Figure 7:**
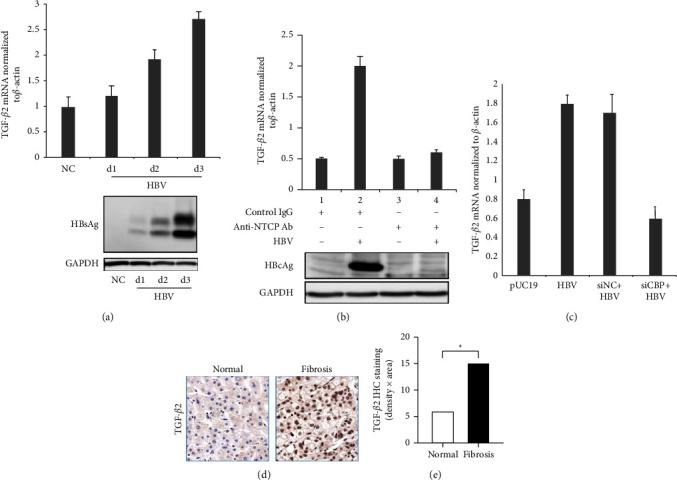
HBV infection up-regulates CREBBP expression and induces high expression of TGF-*β*2. (a)–(c) qRT-PCR was used to detect the mRNA levels of TGF-*β*2 in cells under different treatments. (d) Immunohistochemical staining of TGF-*β*2 in liver biopsy specimens. (e) The semiquantitative score of TGF-*β*2 immunohistochemical staining (*P*  <  0.05).

The TGF-*β*2 expression level gradually increased within 3 days after the HepG2-NTCP cell line was transfected with HBV, and it was positively correlated with HBsAg (Figure 7(a)). After preventing HBV infection, the expression level of TGF-*β*2 will decrease (Figure 7(b)). In addition, HepG2-NTCP cells were transfected with knockdown CREBBP in 24 wells, transfected for 48 hours, infected with HBV at a cell density close to 100%, and then harvested 5 days after infection to measure intracellular TGF-*β*2. The mRNA level is reduced (Figure 7(c)). At the same time, compared with normal liver tissues, TGF-*β*2 is highly expressed in liver cancer tissues associated with liver fibrosis induced by HBV infection (Figures 7(d) and 7(e)). The above results indicate that acetylation of CREB binding protein mediates the expression of TGF-*β*2 induced by HBV infection.

## 4. Discussion

The hepatitis B virus causes liver damage by involving hepatocytes, macrophages, and hematopoietic stem cells in a complex process [19, 20]. Among the various cytokines related to liver fibrosis, TGF-*β* has been proven to be the most important [21]. Previously, it was believed that TGF-*β* can be secreted by hepatocytes and macrophages to activate hepatic stellate cells [22]. Increasing evidence shows that transforming growth factor-*β* mainly transmits signals through Smad [23]. In addition, there are reports that Smad helps *β*-catenin transport to the nucleus of hematopoietic stem cells and initiates the expression of fibrosis genes [24]. The research of antifibrotic drugs is still a top priority. We found that HBV infection can promote the increase of CREBBP acetylation level and highly enrich in TGF beta pathway through acetylation-modified proteomics detection. In recent years, etiological treatment, anti-inflammatory and liver protection, inhibition of hepatic stellate cell activation and proliferation, reduction of excessive production of extracellular matrix, and acceleration of ECM degradation have become important means to inhibit the development of liver fibrosis [25]. However, there is no report on the molecular biology of acetylation-related fibrosis.

Differential proteins are strongly enriched in the “transcriptional coactivator activity” molecular functions in the cluster analysis heat map of GO classification and in the TGF-*β* signaling pathway of the KEGG pathway. The differentially modified proteins are enriched in the CBP/p300 atypical loop domain and CBP/p300 histone acetyltransferase domain. Finally, through cell experiments and immunohistochemistry of liver cancer tissue samples, it was verified that HBV infection caused the increase of TGFbeta expression and the high expression of TGFbeta in liver cancer tissue. However, there is no report on the molecular biology of acetylation-related fibrosis. Our study elucidated the new molecular mechanism of fibrosis-related liver cancer caused by HBV infection from the protein modification level. So far, most antifibrotic drugs are still in the preclinical research stage. In the clinical research stage, there are also some drugs with obvious antifibrotic effects, good safety, and good tolerance. It is believed that with in-depth research on the pathogenesis of liver fibrosis and the continuous advancement of new drug development, it will be possible to reverse liver fibrosis.

## Figures and Tables

**Table 1:** Enrichment of TGF-*β* signaling pathway analysis.

KEGG pathway	Fisher's exact test value	Mapping	Fold enrichment	Protein accession	Position	Ratio	Regulated type	*P*value	Amino acid	Gene name
Hsa04350 TGF-beta signaling pathway	0.001140647	2	36.36	Q09472	1546	2.383	Up	0.00027586	K	EP300
Hsa04350 TGF-beta signaling pathway	0.001140647	2	36.36	Q09472	1549	2.571	Up	0.0098439	K	EP300
Hsa04350 TGF-beta signaling pathway	0.001140647	2	36.36	Q09472	434	2.457	Up	0.0037204	K	CREBBP
Hsa04350 TGF-beta signaling pathway	0.001140647	2	36.36	Q09472	439	2.457	Up	0.0037204	K	CREBBP

## Data Availability

The datasets during the current study are available from the corresponding author on reasonable request.
